# Population-Based Prevalence of Oral Conditions as a Basis for Planning Community-Based Interventions: An Epidemiological Study From Rural Burkina Faso

**DOI:** 10.3389/fpubh.2021.697498

**Published:** 2021-07-01

**Authors:** Alexandra Clauss, Ali Sie, Pascal Zabre, Jörg Schmoll, Rainer Sauerborn, Stefan Listl

**Affiliations:** ^1^Heidelberg Institute of Global Health, Heidelberg University Hospital, Heidelberg, Germany; ^2^Institut National de Santé Publique, Ouagadougou, Burkina Faso; ^3^Radboud Institute for Health Sciences, Radboud University Nijmegen Medical Centre, Nijmegen, Netherlands; ^4^Section for Translational Health Economics, Heidelberg University Hospital, Department of Conservative Dentistry, Heidelberg, Germany

**Keywords:** global oral health, epidemiology, community-based interventions (MeSH), oral health (source: meSH, NLM), universal health coverage

## Abstract

**Objectives:** The purpose of the present study was to: (i) provide timely data on dental caries and periodontal conditions in rural Burkina Faso; (ii) identify the oral health practices carried out in this population, and (iii) to prioritize evidence-based interventions at the community level.

**Methods:** Leaning on WHO recommendations for oral health epidemiological studies, clinical examinations and questionnaire-based surveys were conducted in two different age groups (adolescents: 15–19 years old; adults: 35–44 years old) in the health district of Nouna, Burkina Faso. Caries and tooth status were assessed according to the DMFT Index. The periodontal status was assessed by a modified Community Periodontal Index on all teeth present and measuring the level of attachment-loss of six index teeth. Questionnaire items specifically included utilization of oral health care and oral health behaviors.

**Results:** The prevalence of untreated caries was 38% in adolescents and 73% in adults. In terms of periodontal health, 21% of adolescents and 61% of adults had an attachment loss ≥4 mm. Ninety seven percent of adolescents had not attended a dentist in the previous year and 78% of adults had never seen a dentist in their life. About one third of adolescents and adults cleaned their teeth less often than once per day with equal proportions of toothbrushes and traditional chewing sticks made of tree branches. Fluoride toothpaste was used by <10% of study participants. Almost half of the examined people reported to drink a sugar-sweetened beverage at least once a day.

**Conclusions:** Dental caries and periodontitis are highly prevalent in rural Burkina Faso. These findings highlight the key relevance of epidemiological data for identifying people's oral health needs as basis for developing, testing, and implementing oral health interventions and programs. Special emphasis should be put on the design and evaluation of community-based interventions.

## Introduction

Oral conditions are the most prevalent diseases globally affecting 60–90% of children and adults. They are the third most expensive diseases to treat (1, 2). “Although largely preventable, caries and periodontitis can lead to real pain and suffering, difficulties in eating and chewing, tooth loss, restricted social participation, as well as reduced productivity at work and school.” (3). Alongside many other diseases, dental diseases disproportionally affect low-income populations (4). However, oral health has not been considered an essential component in many health systems worldwide. Universal Health Coverage (UHC) has gained considerable momentum and become a priority in line with the United Nations' sustainable development goals (SDGs) but more efforts are needed to include oral health in UHC (5). While up-to-date reliable data on the epidemiology of caries and periodontitis are critical for designing oral health improvement programs, limited availability of such data, particularly in low resource settings, complicates progress of oral health programs toward UHC.

A previous review of the global epidemiology of caries and periodontitis highlighted the limited evidence on global oral health epidemiology (6). Overall, the prevalence of caries and periodontitis was considered high. However, given the limitations of available evidence, no firm conclusions could be drawn about the incidence and prevalence of periodontitis (6). Not least, the availability of oral health data from Low Income Countries (LICs), particularly Sub-Saharan Africa, is very sparse. Previous meta-regressions from the Global Burden of Disease (GBD) study were based on caries data from 187 countries worldwide (including 12 Sub-Saharan Africa countries) and periodontitis data from 37 countries worldwide [including five Sub-Saharan Africa countries; (7, 8)]. In addition, epidemiological data may be of limited relevance for needs-based planning of oral health programs, if such data does not include all relevant oral health conditions, e.g. periodontitis) or was collected several years ago or was carried out only on facility-based study populations. For Burkina Faso, the latest iteration of the GBD study did not include any input data on periodontitis and the input data on caries did not cover time periods after 2009 (8–12). Such limited availability of epidemiological oral health data substantially hampers the planning of interventions such as community-based oral health programs.

Against this background, the purpose of the present study was to: (i) provide updated data on the prevalence and severity of dental caries and periodontal conditions in a population in rural Burkina Faso; and (ii) identify the oral health practices carried out in this population. The findings from this study were intended (iii), to inform the design and implementation of a community-based intervention, which takes account of the epidemiological data, the specific local context and relevant health systems characteristics in Burkina Faso.

## Materials and Methods

This study was conducted in accordance with the Declaration of Helsinki and approved by the ethical committee of the University of Heidelberg (S-031/2014) and Center de Recherche en Santé de Nouna (CRSN; 2014-01-CIE/CRSN). The reporting follows the STROBE Statement for observational studies.

### Study Setting

The study was conducted in the district of Nouna in Burkina Faso, a country in Sub-Saharan Africa (West). Burkina Faso has a population of more than 18 million people with nearly half of the population living below the income poverty line of US$ 1.25 per day (13). Nouna is a semi-urban town and capital of the Kossi Province in the northwestern part of Burkina Faso. The health care system in Burkina Faso is administered within 13 health regions and 63 health districts. Each district covers a population of 150,000–200,000 people. Across the whole of Burkina Faso with its 18 million inhabitants, 32 dentists provide oral health services together with a larger number of specially qualified dental nurses (14). In the Nouna district, dental health services are integrated into the central hospital (CMA) in Nouna, which is staffed with two nurses, who are specialized in oral health surgery, predominantly tooth extraction. There are no dentists working in the Nouna health district. The nearest dental practices are more than 200 km away, in Kodougou or Bobo-Dioulasso.

### Clinical Examinations and Questionnaires

Based on WHO recommendations for oral health epidemiological studies, clinical examinations and a questionnaire-based survey were conducted in March and April 2014 within two age groups (adolescents: 15–19 years old; adults: 35–44 years old). Using plane mouth mirrors and a WHO CPI ball tip probe, the dental and periodontal status of the person was recorded on a Clinical Record Form (CRF), as recommended by WHO (15). The status of hard tooth tissues was assessed according to the DMFT index, including all permanent and wisdom teeth present [D = Decayed, i.e., caries or filled with caries: codes 1 or 2; F = Filled (no caries), code 3; M = Missing: code 4]. Determination of sound or carious hard tissue followed WHO recommendations (15).

The periodontal status was assessed by a modified Community Periodontal Index (CPI) on all teeth present which also measured the level of attachment-loss of six index teeth. Measurements were conducted on all teeth present on six measuring points with a sensing force of no more than 20 g. Information on loss of attachment (AL) was collected from the index teeth 17/16, 11, 26/27, 37/36, 31 and 46/47 dividing the mouth into sextants. The loss of attachment (AL) was defined as the distance of the cemento-enamel-junction to the bottom of the periodontal pocket which showed cumulative effects of the disease. If the cemento-enamel-junction was not visible, the distance between the free gingival margin and the cemento-enamel-junction was subtracted from the distance between the free gingival margin and the bottom of the periodontal pocket. The most severe finding among the index teeth in each sextant was recorded. If no index tooth was present in a sextant all the teeth present were examined and the highest score recorded. Aiming at broad comparability with earlier and recent periodontal classifications, we synthesized across criteria used by the GBD study, the CPI, and according to the 2017 World Workshop on the Classification of Periodontal and Peri-Implant Diseases and Conditions (7, 16). We accordingly report persons with “severe periodontitis” (GBD criterion: probing depth ≥6 mm; equivalent to CPI score 4), persons with “shallow pockets” (probing depth 4-5 mm = CPI score 3), and persons with attachment loss ≥4 mm as a rough (upper bound) proxy for ‘periodontitis stage III-IV’. The questionnaire was developed in accordance with WHO recommendations with some adaptations to local context and relevant information on access/utilization of oral health care as well as oral health behaviors (15). When a participant did not understand French, the questionnaire was translated into Dioula, the lingua franca in the study area, during the interview.

### Study Personnel and Logistics

The study was coordinated by a formally qualified dentist (AC). Two local dental health workers (Attachés de Santé), registered nurses with specialization in oral surgery, were involved in clinical examinations. Two additional registered nurses of the CMA of Nouna were trained to record study results. Their task was to assist the investigator and follow the examinations attentively to recognize obvious mistakes or omissions. They were also responsible for the arrangement of materials and a sufficient supply of sterile instruments. For the questionnaire-based survey, a team of four interviewers, working for the Center de Recherche en Santé de Nouna (CRSN), was recruited to support the data collection by sensitizing and questioning the participants. For each study day, study personnel were distributed into two field teams, each consisting of a dental nurse, a recording nurse, and an interviewer. Data collection took place in Nouna and larger surrounding villages. Considering the logistic feasibility and an efficient time management of the investigations, participants were requested to assemble in one location. In the town of Nouna, most of the investigations took place in a vacant consultation room of the CMA. For the secluded districts 4 and 7 and the surrounding villages of the HDSS a more proximate location was arranged. Clinical examinations took place inside of a building, assuring the same light conditions for all clinical examinations. An artificial light source was provided for both examiners by a headlamp with a power of 60–80 Lumen (Petzl Tikka XP).

### Training and Calibration of Examiners and Interviewers

Extensive training and calibration were carried out before and during the study. The clinical calibration of the study coordinator (AC) took place at the Department of Conservative Dentistry at the University of Heidelberg, using a calibration method for examiners of the German National Cohort study (17). Training of the two examiners, the two recording assistants and the four interviewers took place at the CRSN in Nouna, Burkina Faso. In advance of the field phase, a 2-day training course for study personnel was organized and held by the study coordinator at the CRSN in Nouna. Clinical examiners were trained “on each other” among the study personnel and in 10 voluntary persons. In addition, a calibration of the investigators took place to assure inter-examiner reliability. The study coordinator and the two examiners evaluated the oral health status of 20 volunteering individuals. Any discrepancies between the results of investigators were reexamined and resolved in group discussions. During the field phase, the number of study participants was distributed equally between the examiners. The study coordinator carried out regular quality checks on clinical assessments. Interviewer training included methodological information and a comprehensive explanation of the structure and the contents of the questionnaire and the completion of the form. A group discussion on the translation of the questions into the widely spoken Dioula took place and a dental terminology was defined. Each interviewer practiced the questionnaire in front of the team and pre-tested it in 10 randomly chosen persons before the beginning of the field-phase.

Appropriate consistency levels were achieved between the study coordinator and the clinical examiners, assuring appropriate inter-examiner reliability. The kappa-value between examiners was 0.9 and the kappa value between study coordinator and examiners was 0.86, indicating strong interrater reliability.

### Sampling, Participant Recruitment, and Data Management

Sample size calculations were informed by previous estimates for the prevalence of caries and periodontitis in Burkina Faso (11). Considering a confidence interval of 95% and an acceptable error margin of ± 5% a total number of 323 examined individuals were required. Considering a cluster effect of 1.3, a total of at least 420 participants had to be examined in each age group. The expected response rate of 80% led to the final sample size including 503 persons each in both age groups. The study sample was drawn from the clustered Nouna Household Panel Survey, which, in turn, is part of the Health and Demographic Surveillance System (HDSS) in Nouna (18). Survey participation was voluntary. Participants were free to refuse their participation at any point of the study (19).

The local distribution between semi-urban and rural inhabitants was taken into consideration to be sampled in proportion with the repartition in the population. Within each age group the repartition of inhabitants of the semi-urban town of Nouna (40%) and the surrounding villages (60%) was represented proportionally. Sensitization of the population and study participants for the study involved communication and announcements via administrative authorities, representatives of Nouna, village informers working for CRSN, and personal introductions of interviewers on the day before the interview. This served to create a climate of confidence and confidentiality among study participants.

To be included in the study, participants had to be a member of a randomly chosen household within the HDSS Household Survey in in Nouna. A member of a household was defined as a member of the basic socio-economic unit of a related or not related family. To determine the different age groups within the sample the year of birth defined the age of a person. Many people living in this remote area of Burkina Faso did not have valid information on their date of birth. Only in a small number of cases the date of birth was based on birth certificates, in other cases the date of birth was deduced through comparison with persons of a similar age or using a 'local events calendar'.

After collection of the information described above, a voluntary consent was attained and recorded before including the person of interest and interviewing or examining it for the study. A person was excluded from the investigations if a severe infection or their general health status hindered the interview or the clinical examination. Information on the medical history was inquired beforehand. Increased risk factors for endocarditis, as an experienced heart infection or a cardiac defect, were criteria of exclusion.

Data cleaning, processing, and descriptive statistical analysis were carried out by the study coordinator (AC), using Microsoft Office Excel (2011) and SPSS (Version 22).

## Results

A total of 827 individuals (including 400 adolescents in the age group 15–19 years and 427 adults in the age group 35–44 years) from the region of Nouna, Burkina Faso, participated in the study during the field phase period of 6 weeks in March and April of 2014. All study participants lived in households of the Nouna Health District and were part of the HDSS. Relative to the target of reaching 503 persons in each age group, this corresponds to a response rate of 80% (adolescents) and 85% (adults), respectively. Detailed demographic characteristics are provided in Table A1.

**Figure 1:**
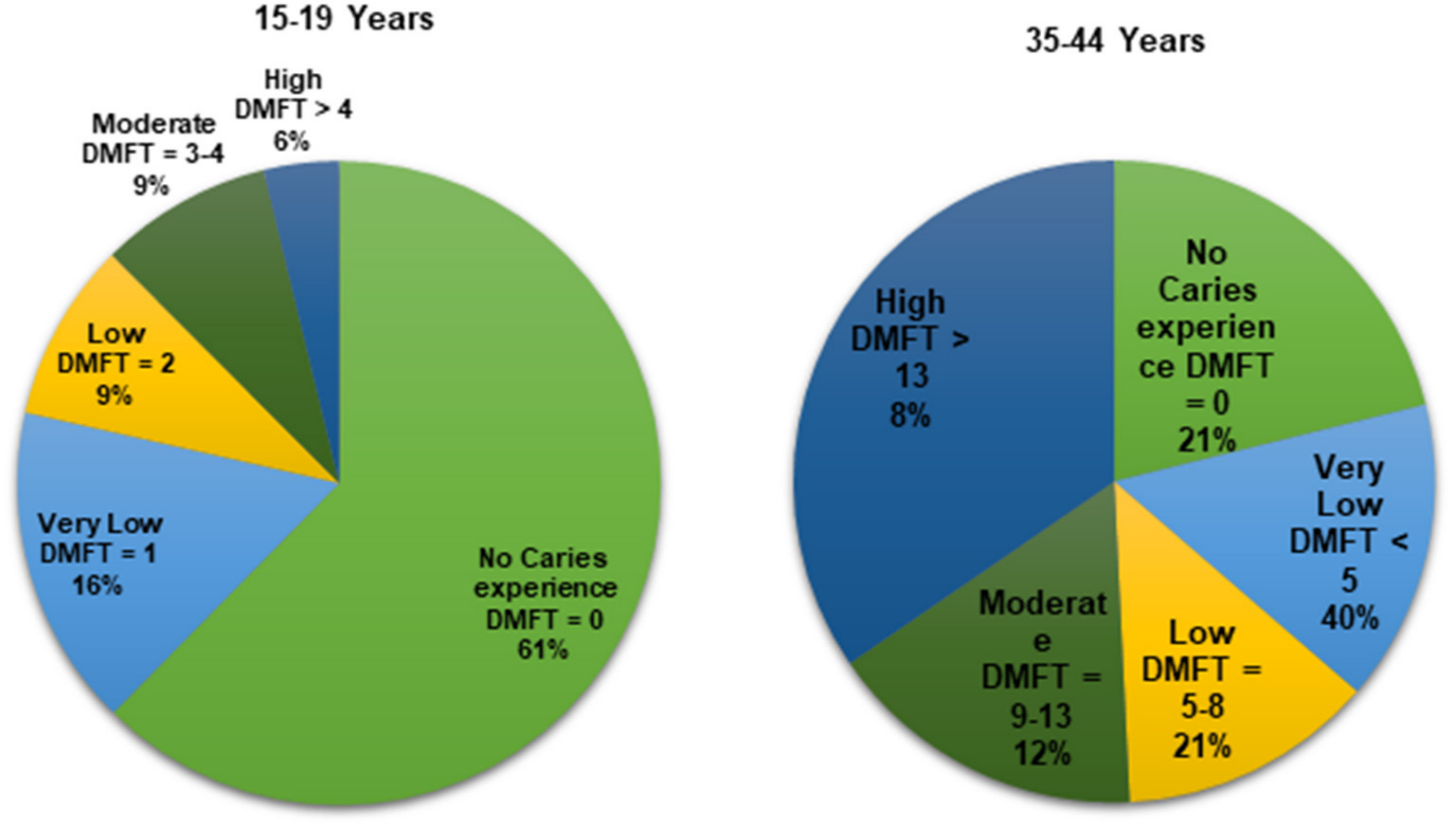
Study participants' DMFT value.

Figure 1 and Table 1 describe people's DMFT values. Among adolescents, mean DMFT was 1.1 (±2.3), and 61% had not experienced any caries. The DMFT burden was largely attributable to decayed teeth (mean DT = 1.0). Sixteen percent had a very low caries experience (DMFT = 1), 9% had low caries experience (DMFT = 2), 9% had moderate caries experience (DMFT = 3–4), and 6% had a high caries experience (DMFT >4). Of the 156 adolescents with caries experience, 153 had untreated decayed teeth, translating to an untreated caries prevalence 38%. Among adults, mean DMFT was 5.0 (±5.7), and 81% had experienced caries in one or more teeth (DMFT ≥1). The DMFT burden was attributable to decayed teeth (mean DT = 3.6) and missing teeth (mean MT = 1.4). Forty percent had a very low caries experience (DMFT <5), 21% a low caries experience (DMFT = 5–8), 12% a moderate caries experience (DMFT = 9–13), and 8% a high caries experience (DMFT >13). Out of the 345 adults with caries experience, 311 had untreated decayed teeth, translating to an untreated caries prevalence of 73%.

**Table 1:** Mean (standard deviation) of DMFT.

**Index**	**15–19 years**	**35–44 years**
DT decayed teeth	1.0 (±2.0)	3.6 (±4.1)
MT missing teeth	0.1 (±0.5)	1.4 (±3.3)
FT filled teeth	-	-
DMFT	1.1 (±2.3)	5.0 (±5.7)

Table 2 describes participants' number of remaining natural teeth and periodontal health status. Only a few people had <20 remaining permanent teeth. Pocket probing teeth was 4–5 mm among 56% and ≥6 mm among 4% of adolescents. Twenty percent of adolescents had an attachment loss ≥4 mm. Pocket probing teeth was 4–5 mm among 67% and ≥6 mm among 13% of adults. Sixty one percent of adults had an attachment loss ≥4 mm.

**Table 2:** Number (%) of individuals by number of teeth and periodontal health status.

	**15–19 years old**	**35–44 years old**
	**number (%)**	**number (%)**
**Permanent teeth**		
≥20	399 (>99%)	420 (98%)
<20	1 (<1%)	7 (2%)
Periodontal health^*^		
Shallow pockets (4–5 mm)	222 (56%)	286 (67%)
Attachment loss ≥4 mm (upper bound proxy for periodontitis stage III–IV)	82 (21%)	259 (61%)
Severe periodontitis (pockets ≥6 mm)	16 (4%)	55 (13%)

* *According to place of greatest score measured in individuals affected*.

Questionnaire results are reported in Table 3. In adolescents, 97% had not attended a dentist in the preceding 12 months. One third of adolescents cleaned their teeth less often than once a day. The most frequently used tooth cleaning products were chewing stick (used by 70% of adolescents) and toothbrush (52%). Fluoride toothpaste was used by 7% of adolescents. During the preceding 12 months, 3% of adolescents had been absent from school due to problems with their teeth. The most prevalent cariogenic food/drinks consumed were tea with sugar (consumed every day by 46% of adolescents) and coffee with sugar (38%). In adults, 78% had never utilized dental care in their life. Over one third of adults cleaned their teeth less often than once a day. The most frequently used tooth cleaning products were chewing stick (used by 73% of adults) and toothbrush (57%). Fluoride toothpaste was used by 10% of adults. During the preceding 12 months, 16% of adults had been absent from work due to problems with their teeth. As was the case for adolescents, the most prevalent cariogenic food/drinks in adults were tea with sugar and coffee with sugar (both consumed by 47% of adults).

**Table 3:** Percentage of individuals by select self-reported items.

	**15–19 years old**	**35–44 years old**
**Dental visit in the past 12 months**		
Never	97%	NA
Once or more	2.5%	NA
Not during the past 12 months	0.8%	NA
Don't know	0.3%	NA
Ever utilized dental care		
No	NA	78%
Yes	NA	22%
<6 months ago	NA	2%
6–12 months ago	NA	4%
1 to 2 years ago	NA	1%
2 to 5 years ago	NA	7%
5 years or longer ago	NA	8%
**Tooth cleaning frequency**		
Never	9%	10%
Once a month	1%	3%
2–3 times a month	2%	2%
Once a week	6%	7%
2–6 times a week	16%	15%
Once a day	51%	54%
Twice or more a day	15%	9%
Product used for tooth cleaning^*^		
(Doesn't clean teeth)	(9%)	(10%)
Chewing Stick	70%	73%
Toothbrush	52%	57%
Fluoride toothpaste	7%	10%
Charcoal	3%	4%
Wooden Toothpick	25%	25%
Plastic Toothpick	3%	1%
**Absent from school/work due to oral problems (last 12 months)**		
Yes	3%	16%
No	97%	84%
Daily consumption of cariogenic foods/drinks^*^		
Fresh fruits	17%	17%
Biscuits, cakes	8%	6%
Sweet pies, buns	1%	<1%
Jam or honey	1%	1%
Chewing gum with sugar	8%	2%
Sweets, candy	7%	6%
Soft drinks	6%	3%
Fruit juice	1%	1%
Tea with sugar	46%	47%
Coffee with sugar	38%	47%

* *Multiple answers possible*.

## Discussion

The findings of the present study suggest that dental caries and periodontitis are highly prevalent health conditions in the examined population in rural Burkina Faso. The values compare high relative to previous GBD estimates for Sub-Saharan Africa (West) which indicated a prevalence of untreated caries in the permanent dentition of about 30% and a prevalence of severe periodontitis of about 10% (6). The reported low access and utilization of oral health care can largely be attributed to the extremely low number of dentists in Burkina Faso and paucity of other types of oral health care. While provision of professional dental care is often considered a sensible strategy for managing oral health in high-income settings, the substantial costs of dental equipment and materials as well as dentist remuneration and training of dentists, most likely are out of the affordable range in settings such as examined in the present study.

In terms of people's oral health preventive behaviors, the study hints at several potential intervention points. First, the frequency of tooth cleaning could be improved in a considerable part of the population. Second, the use of fluoride toothpaste could be increased bearing in mind that WHO recommends fluoride toothpaste as an essential means of oral health prevention (20). However, given that fluoride toothpaste is usually applied with a toothbrush but large parts of the population are using a chewing stick (which is not usually applied with fluoride toothpaste), the combination of tooth cleaning method and fluoride delivery mode should be considered carefully. Third, the intake of cariogenic foods/drinks, such as sugar-sweetened tea or coffee, could be reduced. Sugar consumption might become an even bigger oral health threat for the population in Burkina Faso, considering that the global average per capita consumption of sugar is expected to increase to 24.2 kg/cap by 2,028, with Africa accounting for one of the largest increases in sugar consumption (21).

Drawing on the above findings and leaning on currently available evidence on the effectiveness of oral health interventions, a community-based program to manage oral health could feature the following components (in the sense of an Essential Package of Oral Care to facilitate UHC):

- Training of community health workers to: (i) improve people's personal oral hygiene (tooth cleaning with fluoride application); (ii) reduce people's intake of cariogenic foods/drinks; (iii) screen and detect oral diseases; (iv) provide basic dental treatments (see technologies below); and (v) triage patients with more complex conditions to specialized oral health facilities.
- Caries management: Non-Restorative Treatment and/or Atraumatic Restorative Treatment (22–24).
- Periodontal treatment: given the dearth of evidence on periodontal treatment in community settings, feasibility testing could consider mechanical therapy and/or short-term metronidazole/amoxicillin (22).
- Simple tooth extractions and basic emergency care for acute problems (22).

It is important to note that the effectiveness of any oral health program will also depend on the broader political and economic context as well as human resource planning. First, efforts for the planning of human resources for oral health have been non-existent in many countries or been limited to overly simplistic planning models. For sustainable investment in oral health, recent investment case modeling highlights that careful choices are required about both human resource and treatment technology inputs (25). In line with WHO's global strategy on human resources for health, the relevance and feasibility of oral health needs-based planning of human resources has recently been demonstrated (26). Second, the promotion of reduced sugar intake by community health workers is unlikely to have large effects if, at the same time, sugary products are promoted according to the interests of the sugar industry (27). Third, while fluoride toothpaste is recommended by WHO as an essential health improving product, regulators continue to apply tax rates to fluoride toothpaste as if it was a purely cosmetic product. Fluoride toothpaste may not be affordable for everyone in Burkina Faso (28).

With respect to operationalizing UHC, the findings of the present study provide a number of implications. First, the high prevalence of dental diseases combined with little usage of fluoride toothpaste and frequent consumption of sweetened tea/coffee suggest a high need for public capacity building for oral health promotion and treatment of active disease. Political willingness will be required to prioritize investments in oral health and care. Second, to take account of context-specific needs and preferences, the prioritization of interventions and implementation testing of concrete pathways of care (who gets what and in which circumstances) should take place in close collaboration with all relevant local stakeholders, that is citizens, health professionals, and policy makers. To this end, a community trial with focus on implementation is on the way in the Nouna region. Third, given the nearest dental practices are very far away, integrated planning of human resources and technology for oral health care is critical. Tradeoffs exist between achieving a broad reach of oral health coverage vs. providing a high technical quality of care. While it seems sensible to train and equip community health workers for providing large parts of oral care, it will also be important to strengthen the local capacity for treatment of complex conditions under the supervision by a qualified dentist. Not least, while the data presented in this study may provide useful baseline information to initiate targeted activities toward achieving UHC, a longer-term monitoring and planning framework will be essential to regularly measure population oral health needs and the extent to which the oral health system enables access to essential oral health care for everyone without financial hardship.

Our study has limitations. During the field phase, some difficulties in localizing the sampled population were encountered, especially in the adolescent group which has the highest rate of migration due to searching for higher education, work or getting married. The study was conducted during the dry season, that is, when agricultural activity is low and younger family members move to bigger cities to search for work. The reliance on DMFT has been standard practice in dental epidemiology for many years but the use of DMFT obscures the causes of M, D, and F as well as respective differentiation in severity of disease (29). Related to this, the comparability of studies which use the DMFT requires that examiners be calibrated against the exact same standards for all studies. To this end, some may ask whether the comparably large caries prevalence as reported in the present study might partially be attributable to relatively sensitive caries detection. While careful attention was paid to appropriate calibration of our study's examiners, we cannot fully rule out the possibility of upward bias. Another limitation, while our results are intended to facilitate comparability with various periodontal criteria proposed in the literature, our study took place before availability of criteria recommended by the 2017 World Workshop on the Classification of Periodontal and Peri-Implant Diseases and Conditions (16). In an attempt to retrospectively approximate the stage of periodontitis, we considered attachment loss ≥4 mm (assessed at index teeth) as a rough proxy for “periodontitis stage III–IV.” Note that an attachment loss of 3–4 mm is recommended as a criterion for periodontitis stage II and attachment loss of ≥5 mm as a criterion for periodontitis stage III/IV. Our records neither allowed such categorization of attachment loss nor consideration of other relevant criteria such as radiographic bone loss. Therefore, the values reported in this study may carefully be considered as upper bound estimates of periodontitis stage III-IV as the inclusion of cases with an attachment level of 4 mm implies overestimation. More generally, our results can only be generalized to the respective source population, that is the Nouna Health District. However, considering that the Nouna Health District is typical for non-urban Burkina in terms of dental coverage, household income and education, the external validity of our study may be considered high.

In conclusion, the present study highlights the relevance of sound epidemiological data for identifying people's oral health needs as a first step toward establishing a community-based oral health program in Burkina Faso, as well as targeting health service and resource planning. If the purpose is to incorporate oral health in approaches toward UHC, clear prioritization of oral health services and resource planning according to people's oral health needs is particularly important in low-resource settings. Failure to prioritize needs-based oral health care programs and services such that these can realistically be implemented in the context of available resources, will inevitably result in continuing negligence of oral health problems in the concrete realities in which they occur. Future work on needs based human resource planning as well as oral health investment case modeling is warranted to ensure the right oral health care will be provided by the right people, at the right place, at the right time, to those most in need.

## Data Availability Statement

The raw data supporting the conclusions of this article will be made available by the authors, without undue reservation.

## Ethics Statement

The studies involving human participants were reviewed and approved by Ethical committee of the University of Heidelberg (S-031/2014) and Centre de Recherche en Santé de Nouna (CRSN; 2014-01-CIE/CRSN). Written informed consent to participate in this study was provided by the participants' legal guardian/next of kin.

## Author Contributions

AC, PZ, and JS contributed to conception, design, data collection, interpretation, and critically revised the manuscript. AS contributed to design, interpretation, and critically revised the manuscript. RS contributed to conception, design, interpretation, and critically revised the manuscript. SL contributed to conception, design, interpretation, drafted and critically revised the manuscript. All authors contributed to the article and approved the submitted version.

## Conflict of Interest

The authors declare that the research was conducted in the absence of any commercial or financial relationships that could be construed as a potential conflict of interest.

## Appendix

**Table A1:** Demographic characteristics of the study sample.

	**15-19 years old**	**35-44 years old**	**Total**
	**Mean (%)**	**Mean (%)**	**Mean (%)**
Female	155 (38.8%)	210 (49.2%)	365 (44.1%)
Male	245 (61.3%)	217 (50.8%)	462 (55.9%)
**Geographic location**			
Semi-Urban	161 (40.2%)	155 (36.3%)	316 (38.2%)
Rural	239 (59.8%)	272 (63.7%)	511 (61.8%)
**Ethnicity**			
Dafing/Marka	144 (36.0%)	147 (34.4%)	291 (35.2%)
Bwaba	97 (24.3%)	137 (32.1%)	234 (28.3%)
Mossi	85 (21.3%)	66 (15.5%)	151 (18.3%)
Samo	41 (10.3%)	38 (8.9%)	79 (9.6%)
Peulh	21 (5.3%)	31 (7.3%)	52 (6.3%)
Other	12 (3.0%)	8 (1.9%)	20 (2.4%)
**Religion**			
Muslim	240 (60.0%)	229 (53.6%)	469 (56.7%)
Catholic	115 (28.7%)	135 (31.6%)	250 (30.2%)
Protestant	27 (6.8%)	27 (6.3%)	54 (6.5%)
Animist or other	18 (4.6%)	36 (8.4%)	54 (6.5%)
**Occupation**			
Primary Sector (agriculture)	109 (27.3%)	207 (48.5%)	316 (38.2%)
Secondary Sector (craftsmen)	-	17 (4.0%)	17 (2.1%)
Tertiary Sector (service provider)	1 (0.3%)	25 (5.9%)	26 (3.1%)
Other	1 (0.3%)	24 (5.5%)	25 (3.0%)
Not applicable	289 (72.3%)	154 (36.1%)	443 (53.6%)
**Educational attainment**			
No formal schooling	125 (31.3%)	330 (77.3%)	455 (55.0%)
Primary school or higher	118 (29.5%)	59 (13.8%)	177 (21.4%)
Other	157 (39.3%)	38 (8.9%)	195 (23.6%)
**Socio-economic status (housing, wealth, and possessions)**			
Highest	110 (27.5%)	110 (25.8%)	220 (26.6%)
High	103 (25.8%)	86 (20.1%)	189 (22.9%)
Mean	67 (16.8%)	84 (19.7%)	151 (18.3%)
Low	74 (18.5%)	84 (19.7%)	158 (19.1%)
Lowest	46 (11.5%)	63 (14.8%)	109 (13.2%)
**Total**	**400 (100.0%)**	**427 (100.0%)**	**827 (100.0%)**
